# Correction: Whole mount multiplexed visualization of DNA, mRNA, and protein in plant-parasitic nematodes

**DOI:** 10.1186/s13007-025-01409-1

**Published:** 2025-07-01

**Authors:** Alexis L. Sperling, Sebastian Eves-van den Akker

**Affiliations:** Department of Plant Sciences, Crop Science Centre, University of Cambridge, 93 Lawrence Weaver Road, Cambridge, Cambridge, CB3 0LE UK

**Correction: Plant Methods (2023) 19:139** 10.1186/s13007-023-01112-z

Following publication of the original article [1], the authors would like to correct the value in the paragraph “Cuticle permeabilization with the Sperling preparation”.

Incorrect value: “5% Sodium dodecyl sulfate (SDS)”

Correct value: “0.5% Sodium dodecyl sulfate (SDS)”
