# Supplementary figures and images for: Whole mount multiplexed visualization of DNA, mRNA, and protein in plant-parasitic nematodes
Source: Plant Methods. 2023 Dec 4;19:139. doi: 10.1186/s13007-023-01112-z (PMC10696717; doi:10.1186/s13007-023-01112-z)

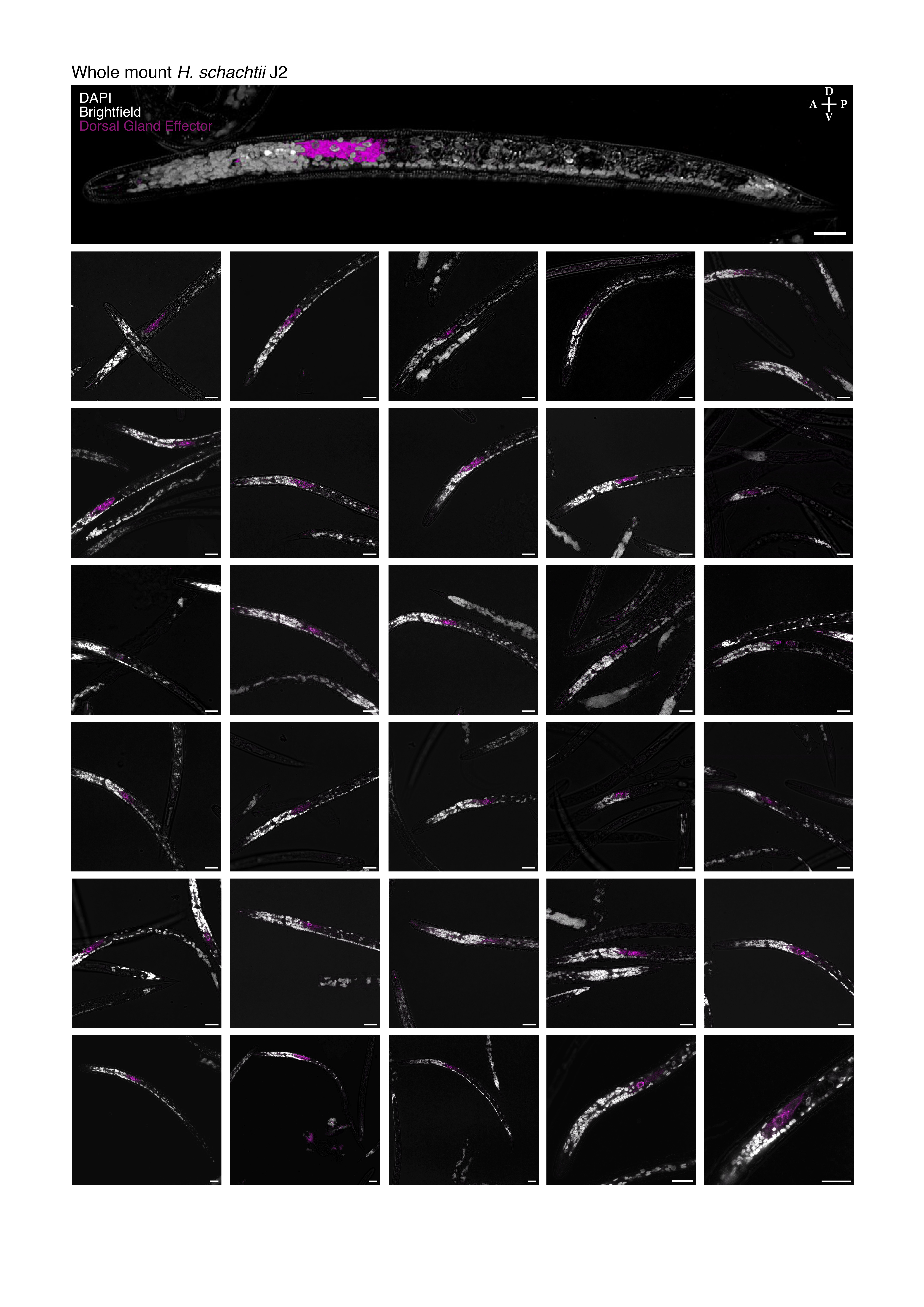

Supplement: Supplementary file 1 — Supplementary Material 1 [file 13007_2023_1112_MOESM1_ESM.jpg]
